# The use of platelet-rich fibrin combined with periodontal ligament and jaw bone mesenchymal stem cell sheets for periodontal tissue engineering

**DOI:** 10.1038/srep28126

**Published:** 2016-06-21

**Authors:** Zhong-Shan Wang, Zhi-Hong Feng, Guo-Feng Wu, Shi-Zhu Bai, Yan Dong, Fa-Ming Chen, Yi-Min Zhao

**Affiliations:** 1 State Key Laboratory of Military Stomatology & National Clinical Research Center for Oral Diseases & Shaanxi Key Laboratory of Oral Diseases, Department of Prosthodontics, School of Stomatology, Fourth Military Medical University, Xi’an, Shaanxi, China; 2 State Key Laboratory of Military Stomatology & National Clinical Research Center for Oral Diseases & Shaanxi Engineering Research Center for Dental Materials and Advanced Manufacture, Department of Periodontology, School of Stomatology, Fourth Military Medical University, Xi’an, Shaanxi, China

## Abstract

Periodontal regeneration involves the restoration of at least three unique tissues: cementum, periodontal ligament tissue (PDL) and alveolar bone tissue. Here, we first isolated human PDL stem cells (PDLSCs) and jaw bone mesenchymal stem cells (JBMSCs). These cells were then induced to form cell sheets using an ascorbic acid-rich approach, and the cell sheet properties, including morphology, thickness and gene expression profile, were compared. Platelet-rich fibrin (PRF) derived from human venous blood was then fabricated into bioabsorbable fibrin scaffolds containing various growth factors. Finally, the *in vivo* potential of a cell-material construct based on PDLSC sheets, PRF scaffolds and JBMSC sheets to form periodontal tissue was assessed in a nude mouse model. In this model, PDLSC sheet/PRF/JBMSC sheet composites were placed in a simulated periodontal space comprising human treated dentin matrix (TDM) and hydroxyapatite (HA)/tricalcium phosphate (TCP) frameworks. Eight weeks after implantation, the PDLSC sheets tended to develop into PDL-like tissues, while the JBMSC sheets tended to produce predominantly bone-like tissues. In addition, the PDLSC sheet/PRF/JBMSC sheet composites generated periodontal tissue-like structures containing PDL- and bone-like tissues. Further improvements in this cell transplantation design may have the potential to provide an effective approach for future periodontal tissue regeneration.

Periodontitis, a widespread infectious disease in humans^1^, is the main cause of tooth loosening and loss, which lead to the progressive destruction of tooth-supporting tissues, including the alveolar bone, periodontal ligament (PDL) and root cementum^1^,^2^. In addition, periodontitis has been associated with a number of systemic diseases, including diabetes mellitus, cardiovascular disease and stroke^3^. Conventional clinical treatment strategies that include tooth scaling, root planing and open-flap debridement can effectively eliminate pathogenetic agents and promote tissue self-repair^4^. Other regenerative therapies, such as guided tissue regeneration, bone grafting and enamel matrix derivative product application, have routinely been used in clinical practice to guide periodontal tissue regeneration. However, the outcomes of these therapies have been limited because they have failed to consistently restore the complete periodontium, which comprises a complex structure that includes the PDL, cementum and alveolar bone^4^,^5^.

Stem cell-based periodontal regeneration is being developed rapidly, and dental stem cells, such as PDL stem cells (PDLSCs)^6^,^7^, dental follicle cells (DFCs)^8^, and dental pulp stem cells^9^, are increasingly being investigated as easily accessible undifferentiated cells. Non-dental stem cells, such as bone marrow-derived mesenchymal stem cells (BMMSCs)^10^, alveolar periosteal cells (APCs)^11^, adipose-derived stem cells^12^ and embryonic stem cells^13^, exhibit the potential for multilineage differentiation to generate replacement tissue. Among these cell types, PDLSCs and DFCs have been widely used for periodontal regeneration in animal studies, and the outcomes have been predictable^6^,^14^,^15^. BMMSCs and APCs have also been reported to successfully differentiate into a PDL^16^. However, in heterotopic implantation nude mice models, PDLSCs tend to differentiate into cementoblast-like cells and to develop cementum-/PDL-like tissues *in vivo*. By contrast, BMMSCs tend to form bone-like tissue and a small amount of fibrous structure^17^. Currently, it is generally accepted that MSCs tend to form new tissues at ectopic sites that correspond to their origin^18^. The complex structure of the periodontium, which comprises both hard (cementum and alveolar bone) and soft (PDL) tissues has made it difficult to determine whether a single progenitor cell type can regenerate the entire structure of the periodontal complex^18^. Therefore, the combined use of PDLSCs and BMMSCs may be an appropriate strategy for periodontal complex reconstruction.

In recent years, cell sheet engineering has emerged as a novel approach for the effective delivery of seeded cells while preserving cell–cell junctions and supplying an *in vitro*-secreted extracellular matrix (ECM) to promote tissue regeneration^19^. This type of cell engineering could effectively preserve the biological and mechanical properties of the cellular microenvironment that are established during *in vitro* expansion, thereby increasing the cell survival rate and reducing cell loss during cell sheet implantation^20^. This technique has also been demonstrated to be effective in the treatment of myocardial infarction^21^, corneal dysfunction^22^ and esophageal ulceration^23^. In this study, we isolated human PDLSCs and jaw bone MSCs (JBMSCs) and then developed these cells into PDLSC and JBMSC sheets for periodontal complex regeneration.

Based on the need for a specific bioabsorbable scaffold for the delivery of therapeutic cell sheets that would improve clinical efficacy and support and sustain cell sheets within the space between the alveolar bone and the root cementum (specifically, PDLSC sheets adjacent to the dentin surface and JBMSC sheets adjacent to the alveolar bone surface), human platelet-rich fibrin (PRF) combined with various growth factors extracted from whole blood was used in this study^24^. This mixture has several advantages over platelet-rich plasma, which has traditionally been used. These advantages include one-step preparation and the production of natural blood products in the absence of anticoagulants^25^. Most importantly, PRF possesses an optimal three-dimensional (3-D) structure that favors the delivery and support of cell sheets at a specific area that has been destroyed^26^,^27^. Because PRF has been used for periodontal tissue healing and has been tested for avulsed tooth reimplantation^24^,^26^, the combined application of PDLSC sheets, PRF and JBMSC sheets may be a more effective approach for periodontal complex regeneration.

In this study, we first isolated human PDLSCs and JBMSCs and compared their differentiation properties. Next, we fabricated PDLSC and JBMSC sheets using an ascorbic acid-rich approach to more effectively load seeded cells and investigated their characteristics, including morphology, thickness and gene expression profile. We then fabricated PRF into a growth factor-rich scaffold, and treated dentin matrix (TDM)^15^ and hydroxyapatite (HA)/tricalcium phosphate (TCP) frameworks^6^ were produced to simulate the interfaces of dentin and alveolar bone, respectively, in a nude mouse implantation model. Finally, PDLSC sheet/PRF/JBMSC sheet composites were loaded into the simulated periodontal space formed by the TDM and HA/TCP frameworks, and the entire transplants were implanted into nude mice for eight weeks to test the hypothesis that this method induces periodontal regeneration. We hope that this cell transplantation method will be a new approach to periodontal regeneration.

## Results

### Isolation and characterization of PDLSCs and JBMSCs

**Figure 1:**
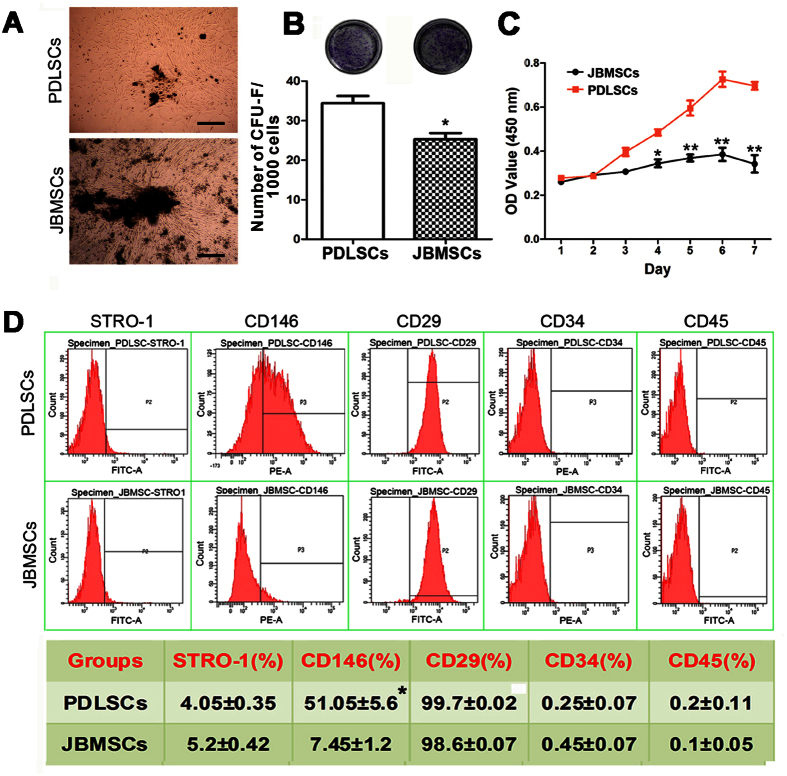
The differences in proliferative capacity and immunophenotype between human periodontal ligament (PDL) stem cells (PDLSCs) and human jaw bone mesenchymal stem cells (JBMSCs). (**A**) The initial PDLSCs and JBMSCs were cultured in six-well dishes containing complete α-MEM. (**B**) Representative images of crystal violet staining of the colonies of PDLSCs and JBMSCs and a comparison of the number of colonies formed by PDLSCs and JBMSCs (*P < 0.05 indicates a significant difference compared with PDLSCs). (**C**) Growth curves for PDLSCs and JBMSCs, as determined by a cell counting kit-8 (CCK-8) assay (*P < 0.05 and **P < 0.01 indicate significant differences compared with PDLSCs at the same time point). (**D**) Flow cytometric analysis indicated that the PDLSCs and JBMSCs were positive for mesenchymal-associated markers CD29, CD146 and STRO-1 but negative for hematopoietic markers CD34 and CD45 (*P < 0.05 indicates a significant difference compared with JBMSCs).

After 5 to 7 days in a primary culture, PDLSCs and JBMSCs successfully proliferated and formed cells that displayed spindle-shaped morphologies and single-cell clones of varying sizes (Fig. 1A). The proliferation capacity of PDLSCs and JBMSCs was compared based on colony-forming unit-fibroblast (CFU-F) assays and growth curve assessments (cell counting kit-8 (CCK-8) assays). Both putative stem cell types demonstrated colony formation ability, although the number of CFU-Fs formed by PDLSCs was higher than that formed by JBMSCs (*P* < 0.01) (Fig. 1B). Similarly, the slope of the cell growth curve following a 7-day incubation was generally much steeper for PDLSCs than for JBMSCs, and the cell number in each group peaked on day 6. Significant differences in cell proliferation were detected between these two putative stem cell types on days 4, 5, 6 and 7 (*P* < 0.01) (Fig. 1C).

### Immunophenotype determination

The immunophenotypes of PDLSCs and JBMSCs were determined using flow cytometry. In general, both putative stem cell types were negative for hematopoietic markers CD34 and CD45 and positive for mesenchymal-associated marker CD29. In addition, PDLSCs and JBMSCs expressed STRO-1 and CD146, both of which are early markers of MSCs. The rate of CD146 positivity was higher among PDLSCs (*P* < 0.05), although no significant difference in the frequency of expression of the other positive or negative markers was observed between these two cell types (*P* > 0.05) (Fig. 1D).

### Osteogenic and adipogenic differentiation *in vitro*

**Figure 2:**
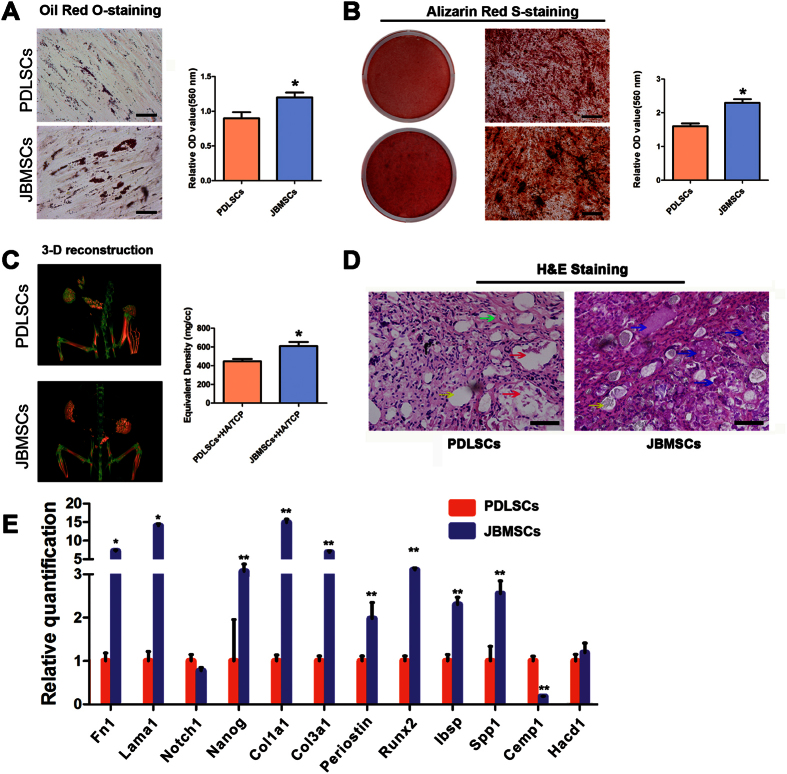
The differences in multi-directional differentiation potential between PDLSCs and JBMSCs. (**A**) Representative images showing the differences in multipotent differentiation between the two cell types. Lipid vacuoles of different sizes and numbers were observed after 2 weeks of adipogenic induction (Oil Red O staining, scale bar = 200 μm). Data analysis was performed on the relative level of Oil Red O dye absorption between PDLSCs and JBMSCs (*P < 0.05 indicates a significant difference compared with PDLSCs). (**B**) Mineralized nodules of different sizes and numbers were formed after 4 weeks of osteogenic induction (Alizarin Red S staining, scale bar = 100 μm). Data analysis was performed on the relative level of Alizarin Red S dye absorption between PDLSCs and JBMSCs (*P < 0.05 indicates a significant difference compared with PDLSCs). (**C**) The difference in osteogenic differentiation capacity between PDLSCs and JBMSCs *in vivo* at eight weeks post-implantation. The three-dimensional (3-D) reconstruction images of grafts were obtained via micro-CT, and the results indicated that the JBMSC/HA/TCP group (more red areas) displayed a higher density than did the PDLSC/HA/TCP group (more green areas). The equivalent bone density was quantitatively analyzed through statistical analysis (*P < 0.05 indicates a significant difference compared with the PDLSC/HA/TCP group). (**D**) Hematoxylin and eosin (H&E) staining of the sections after decalcification showed that the PDLSC/HA/TCP group produced predominantly PDL-like structures (green arrow) and some cementum-like structures (red arrows). By contrast, the JBMSC/HA/TCP group displayed predominantly bone-like structures that varied in appearance and size (blue arrows). The mixed HA/TCP clumps are indicated by yellow arrows in both groups. (**E**) The differences in gene expression between PDLSCs (P2) and JBMSCs (P2) (*P < 0.05 and **P < 0.01 indicate significant differences compared with PDLSCs).

PDLSCs and JBMSCs (P3) were induced in osteogenic or adipogenic media for several weeks to evaluate their multipotent differentiation potential. In terms of adipogenic differentiation, microscopic Oil Red O-positive lipid droplets were observed in both PDLSCs and JBMSCs after 2 weeks of induction. However, more and larger lipid droplets tended to develop in the JBMSCs, and quantitative analysis showed that the optical density (OD) was higher in JBMSCs than in PDLSCs (*P* < 0.05) (Fig. 2A). In terms of osteogenic differentiation, both PDLSCs and JBMSCs formed Alizarin Red S-positive mineralized nodules after 4 weeks of incubation. However, JBMSCs appeared to accumulate more calcium deposits, and statistical analysis showed that the number of mineralized nodules was greater in JBMSCs than in PDLSCs (*P* < 0.05) (Fig. 2B). These results indicated that JBMSCs exhibited higher osteogenic potential than did PDLSCs.

### Osteogenic differentiation *in vivo*

To validate the osteogenic differentiation capacity of PDLSCs and JBMSCs *in vivo*, 3-D reconstruction images of the grafts were obtained eight weeks post-implantation using micro-CT scanning data. These images showed that the JBMSC sheet/HA/TCP group (more red areas) displayed a higher density than did the PDLSC sheet/HA/TCP group (more green areas). Quantitative analysis revealed that the equivalent bone density of the JBMSC sheet/HA/TCP group was higher than that of the PDLSC sheet/HA/TCP group (*P* < 0.05) (Fig. 2C). Hematoxylin and eosin (H&E) staining of the sections revealed that the PDLSC sheet/HA/TCP group contained many collagenous fibers similar to those found in the PDL and few small bone-like structures; by contrast, the JBMSC sheet/HA/TCP group contained many bone-like structures that varied in appearance and size but no evident collagenous fibers (Fig. 2D).

### Gene expression profiles of PDLSCs and JBMSCs

The expression levels of eleven genes related to cellular stemness, adhesion, differentiation and migration were analyzed via real-time PCR. The results indicated that the gene expression profiles of PDLSCs and JBMSCs (P2) were very different. In general, JBMSCs (P2) exhibited higher expression of stemness-related genes (Nanog); adhesion-related genes (fibronectin 1 (Fn1) and laminin subunit alpha 1 (Lama1)); and calcification-related genes (collagen type I, alpha 1 (Col1a1), collagen type III, alpha 1 (Col3a1), Periostin, integrin binding sialoprotein (Ibsp), secreted phosphoprotein 1 (Spp1) and runt-related transcription factor 2 (Runx2)), except for cementum protein 1 (Cemp1), to varying degrees compared to PDLSCs (P2) (Fig. 2E).

### Characterization of cell sheets

**Figure 3:**
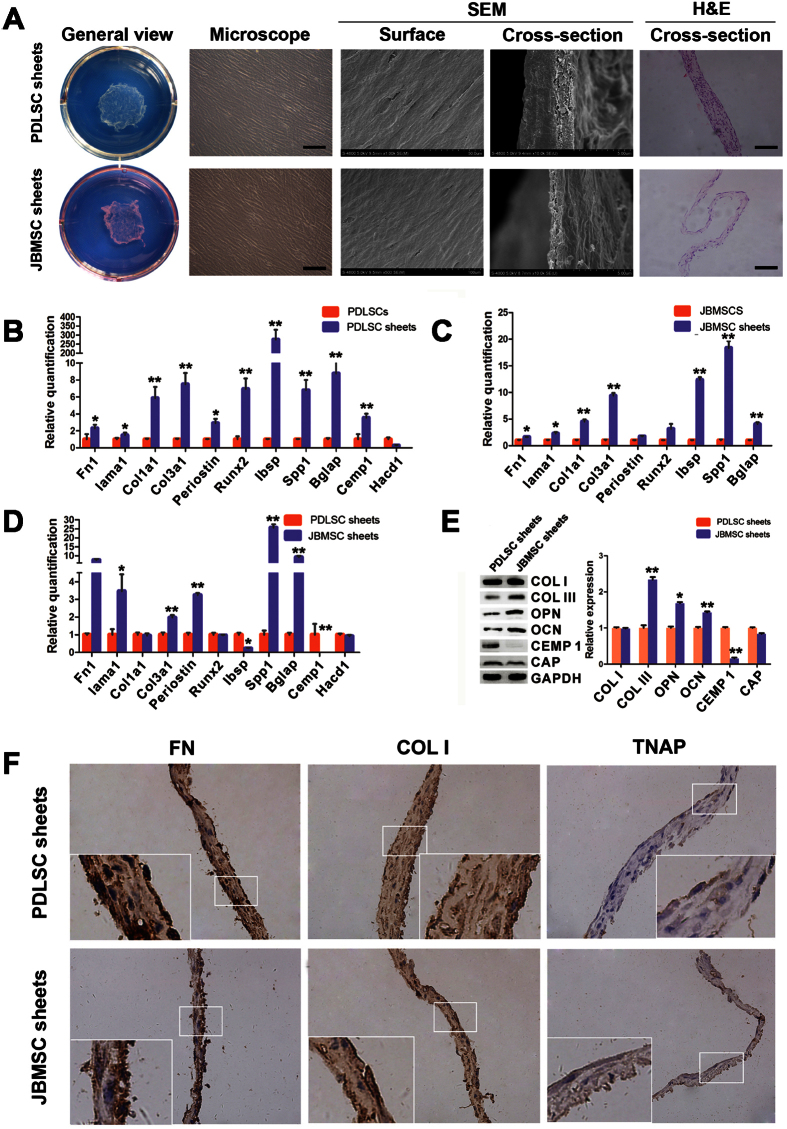
Characterization of the PDLSC and JBMSC sheets. (**A**) Representative images of PDLSC and JBMSC sheets formed in culture dishes (general view), as observed under an inverted microscope (cell arrangement, scale bar = 100 μm), scanning electron microscopy (SEM; cell arrangement and connections), and H&E staining (cross-section, scale bar = 10 μm). (**B**) The relative mRNA expression profiles of PDLSC sheets compared to PDLSCs (*P < 0.05 and **P < 0.01 indicate significant differences compared with PDLSCs). (**C**) The relative mRNA expression profiles of JBMSC sheets compared to JBMSCs (*P < 0.05 and **P < 0.01 indicate significant differences compared with JBMSCs). (**D**) The relative mRNA expression profiles of JBMSC sheets compared to PDLSC sheets (*P < 0.05 and **P < 0.01 indicate significant differences compared with PDLSC sheets). (**E**) The expression levels of periodontal tissue-specific proteins (COL I and COL III), calcification-related proteins (OPN and OCN), and cementum tissue-related proteins (CEMP1 and CAP) in PDLSC and JBMSC sheets as determined by quantitative western blot analysis (*P < 0.05 and **P < 0.01 indicate significant differences compared with PDLSC sheets). (**F**) Immunohistological characteristics of PDLSC and JBMSC sheets. Both types of cell sheets displayed strong positive immunohistochemical staining using anti-human FN1 and anti-human COL I antibodies. Both sheets also displayed weakly positive staining for TNAP.

Cell sheets were formed after a 14-day induction period (Fig. 3A). The cells became confluent and formed film-like cell sheets that could be completely detached from the edges of the dishes. Specifically, the JBMSC sheets appeared softer and thinner than the PDLSC sheets based on gross observation. Using an inverted microscope, we observed that the PDLSC and JBMSC sheets were arranged in a unidirectional or whirlpool-like pattern. Surface observation using scanning electron microscopy (SEM) showed that both types of cell sheets contained many layers of cells that were closely arranged in an orderly pattern. However, based on the observation of lateral sections via SEM and H&E staining, we found that PDLSC sheets contained multiple layers of cells (3 to 7 layers), secreted a rich ECM and tended to form a tight network of collagen fibers that retained tight junctions. The JBMSC sheets contained fewer layers of cells (2 to 4 layers) and secreted less ECM than did the PDLSC sheets. Quantitative analysis showed the thickness of the PDLSC sheets to be significantly greater than that of the JBMSC sheets (*P* < 0.05).

### Gene and protein expressions after cell sheet formation

Altered gene expression between the cells (P2) and cell sheets was determined with real-time PCR. In general, the relative mRNA expression profiles of PDLSCs and JBMSCs were very different after forming cell sheets, and the expression of nearly every gene we evaluated displayed an increasing trend after cell sheet formation. Compared to PDLSCs, PDLSC sheets exhibited higher expression of periodontal tissue-specific genes (Col1a1, Col3a1 and Periostin); calcification-related genes (Runx2, Ibsp, Spp1 and Bglap); and Cemp1 as well as slightly higher expression of adhesion-related genes (Fn1 and Lama1) (Fig. 3B). Compared to JBMSCs, JBMSC sheets displayed higher expression of calcification-related genes (Runx2, Ibsp, Spp1 and Bglap) and periodontal tissue-specific genes (Col1a1 and Col3a1) as well as slightly higher expression of adhesion-related genes (Fn1 and Lama1) (Fig. 3C). We compared the relative gene expression profiles of PDLSC and JBMSC sheets, and the results indicated that compared to PDLSC sheets, JBMSC sheets exhibited increased expression of calcification-related genes (Spp1 and Bglap), adhesion-related genes (Fn1 and Lama1) and periodontal tissue-specific genes (Col3a1 and Periostin); similar expression of Col1a1 and Runx2; and no expression of Cemp-1 (Fig. 3D). We also compared the relative protein expression profiles of PDLSC and JBMSC sheets using western blotting (Fig. 3E), and these results showed higher expression of calcification-related proteins (collagen type 3 (COL III), osteopontin (OPN) and osteocalcin (OCN)) in JBMSC sheets and comparable expression of collagen type 1 (COL I) and the cementum tissue-specific cementum attachment protein (CAP) between the two.

### Immunohistochemical analyses of PDLSC and JBMSC sheets

Immunohistochemical staining (Fig. 3F) showed strong positive staining for COL I and fibronectin 1 (FN1), which are primarily found in ECM and play important roles in maintaining biological functions, in both PDLSC and JBMSC sheets. The expression of alkaline phosphatase (TNAP), an early marker of osteoblast differentiation, was observed as weakly positive staining in both JBMSC and PDLSC sheets. PBS was substituted for the primary antibody as a negative control.

### Characterization of transplanted biomaterials

#### Characterization of PRF

**Figure 4:**
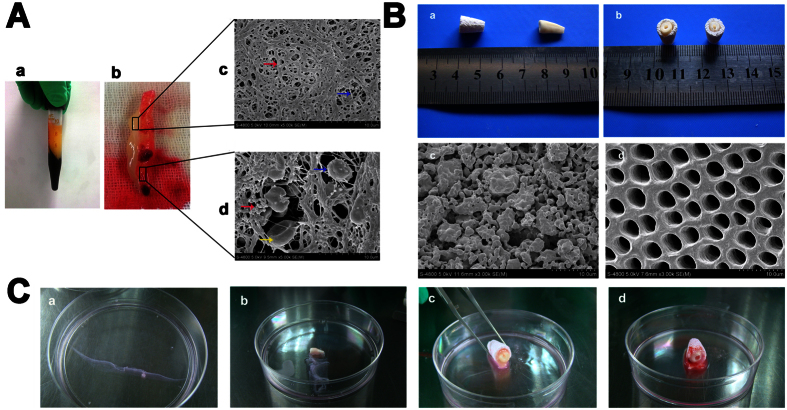
Characterization of transplanted biomaterials (platelet-rich fibrin (PRF), human treated dentin matrix (TDM) and HA/TCP frameworks) and their combined application for periodontal regeneration in a nude mouse model. (**A**) (a,b) PRF clot after centrifugation. (c,d) The ultrastructure of the PRF as observed by SEM. In the top region (c) of the PRF, the 3-D network predominantly comprised fibrin fibers (red arrow) and a few fibrillae with smaller diameters (blue arrow), and few platelets or cells were detected. In the lower region of the PRF (d), numerous platelets (red arrow) and leukocytes (blue arrow) and some red blood cells (yellow arrow) were embedded in the network. (**B**) The macroscopic and microscopic appearance of the human TDM and HA/TCP scaffolds. (a,b) The macroscopic appearance of a TDM and an HA/TCP scaffold with a length of 1.0 cm. TDM was fabricated using a previously described method to simulate dentin, and HA/TCP frameworks with a tooth root-like shape were used to simulate alveolar bone. (c) The appearance of the HA/TCP frameworks of varying size as observed by SEM. The HA/TCP particles were loosely and irregularly arranged, and there were many pores between the particles. (d) The appearance of TDM as dentinal tubules arranged in an orderly manner as observed by SEM. (**C**) (a) Cell sheets were detached after maturation and wrapped in eight layers on the surfaces of the TDM (b). A ring-shaped interspace was formed after insertion of the TDM into the HA/TCP framework (c), and this void provided space for transplanted cell sheets and PRF (d).

PRF containing absorbable fibrin and various human-derived growth factors was employed as a biological scaffold material (Fig. 4A). PRF clots were easily produced after centrifugation and were transformed into resistant fibrin membranes by compressing the fluids out of the fibrin matrix. SEM observation indicated that the PRF microstructure included abundant fibrin fibers and a few fibrillae with smaller diameters that comprised a 3-D network. The upper white region of the PRF exhibited a neat appearance and contained no cells, while the lower red region of the PRF contained many platelets, some leukocytes and few red blood cells, all of which were embedded in the network.

ELISAs were performed to analyze the sequential profile of released growth factors in one complete PRF membrane over 7 days. The results showed that the PRF membrane released growth factors in a time-dependent manner throughout the experimental period (Table 1).

#### Characterization of TDM and HA/TCP frameworks

Human TDM was fabricated using a previously described method to simulate dentin, which was produced in dimensions of 1.0 cm in length. After treatment with EDTA, the dentinal tubules demonstrated an orderly arrangement, as shown by SEM observation of their surface topography. The HA/TCP framework, which was used to simulate alveolar bone, was fabricated in a tooth root-like conformation in which HA/TCP particles of varying size were loosely and irregularly arranged, and numerous pores were present between the HA/TCP particles, as shown by SEM (Fig. 4B).

#### Assessment of new tissues formed *in vivo*

We designed a new method to engineer multiple periodontal tissues and tested its feasibility in a nude mouse model. As shown in Fig. 4, PRF containing absorbable fibrin and various human-derived growth factors was employed as a biological scaffold material (Fig. 4A). TDM was used to simulate the microenvironment of dentin tissue, and HA/TCP frameworks were employed to simulate alveolar bone tissue. In addition, a ring-shaped interspace was formed after inserting the TDM into the HA/TCP frameworks, and this void offered a space for the transplantation of PRF-cell sheet constructs (Fig. 4B). PDLSC and JBMSC sheets supplied seeded cells for PDL and bone tissue engineering, respectively, and PRF membranes were placed between the cell sheets to act as an absorbable biomaterial and provide various native growth factors for new tissue regeneration (Fig. 4C).

**Figure 5:**
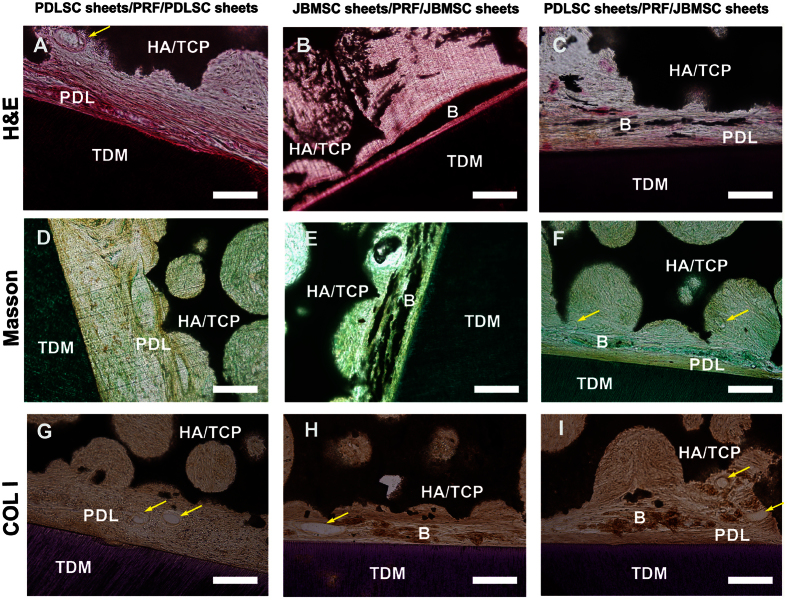
Mimicking periodontal regeneration in a nude mouse model using a PRF scaffold combined with PDLSC and JBMSC sheets. Representative images of periodontal tissue regeneration in the different groups. In group 1 (PDLSC sheets/PRF/PDLSC sheets), abundant collagen fibers (PDL) were produced, and blood vessels (yellow arrows) crossing through the tissue were present, but there was no evidence of bone-like tissue formation. In group 2 (JBMSC sheets/PRF/JBMSC sheets), abundant bone-like tissues (**B**) as well as some newly formed vessels (yellow arrows) were observed; however, fewer collagen fibers were present in group 2 than in group 1. In group 3 (PDLSC sheets/PRF/JBMSC sheets), both PDL- and bone-like structures were produced. Briefly, a dense layer of connective tissue covered the TDM surface. Outside the PDL-like tissue, several layers of bone-like tissue (**B**) were observed (scale bar = 100 μm).

Eight weeks after subcutaneous implantation into nude mice, PDLSC and JBMSC sheets had produced very different structures. Histological observation (Fig. 5) showed that in group 1 (PDLSC sheets/PRF/PDLSC sheets), many identical collagen fibers had formed on the surfaces of both the TDM and HA/TCP frameworks, although these fibers were not obviously inserted and lacked PDL orientation. In group 2 (JBMSC sheets/PRF/JBMSC sheets), abundant bone-like tissues were observed between the TDM and HA/TCP frameworks. By contrast, fewer collagen fibers were detected in group 2 than in group 1. In group 3 (PDLSC sheets/PRF/JBMSC sheets), a dense layer of connective tissue covered the TDM surface. Outside these PDL-like tissues, several layers of bone-like tissues were observed (but not well characterized). In all groups, blood vessel formation was observed within these newly formed PDL-like fibers and bone-like tissues, but we did not detect any obvious cementum-like structures. These findings suggest that PDLSC sheets tend to form fiber tissue, while JBMSC sheets are more likely to form new bone, and the combination of JBMSC and PDLSC sheets may achieve a balance between newly formed PDL- and bone-like tissues in future periodontal tissue engineering applications.

## Discussion

Previous strategies for the restoration of periodontal structures have been based on anti-infectious therapies and have primarily comprised scaling, open flap debridement, guided tissue regeneration, and local administration of anti-inflammatory drugs or various growth factors^2^,^28^. However, careful histological evaluation indicated that these procedures have limited potential for regenerating the PDL or cementum, particularly new alveolar bone^4^. Therefore, approaches using stem cells for periodontal regeneration have recently been advocated. Specific phenotypic stem cells are known to exhibit a tendency to develop new tissues that match their origins in terms of structure and function^17^,^18^. In this study, we isolated two specific phenotypic stem cells, PDLSCs and JBMSCs, for use as seeded cells and compared the differentiation tendencies of these cell types *in vitro* and *in vivo*. To maximally preserve the ECM and the mechanical properties of the cells, reduce cell loss, increase the cell survival rate, and develop an easy and feasible clinical application^26^, we induced these two stem cell types to form cell sheets. We then compared their characteristics and established their differentiation tendencies. To improve the therapeutic efficiency of cell sheets, we placed PRF, which comprises a 3-D fibrin network and produces various growth factors that nourish, support and sustain cell sheets, in the space between the alveolar bone and the root surface^26^. For the *in vivo* model of the implantation of cell sheets and PRF, we designed TDM and HA/TCP frameworks to simulate the interfaces of the root surface and alveolar bone, respectively. Eight weeks after implantation, the PDLSC sheet/PRF/JBMSC sheet composites had achieved a balance between newly formed PDL- and bone-like tissues to form a periodontal tissue-like structure.

First, we compared the characteristics of isolated human PDLSCs and JBMSCs. Although both PDLSCs and JBMSCs showed a spindle-like morphology and positive expression of early markers of MSCs, such as STRO-1 and CD146^29^, a series of assays revealed that these two cell types exhibited very different characteristics. PDLSCs showed more robust proliferation capacity than did JBMSCs because PDLSCs possessed a stronger colony formation ability and a higher cell growth rate, and these results were in line with previous reports^29^,^30^. This finding suggested that during *in vitro* expansion, JBMSCs may take longer to generate seeded cells than do PDLSCs. Therefore, this difference in proliferation should be considered when coordinating the tissue regeneration process^29^. JBMSCs also exhibited higher adipogenic and osteogenic potential than did PDLSCs, and the *in vivo* differentiation experiments demonstrated that the JBMSC sheet/HA/TCP group developed predominantly bone-like tissues^17^. Because multilineage differentiation potential has been considered a key factor in stem cell-mediated tissue regeneration and because several studies have revealed a difference in differentiation potential between stem cell types, this aspect of tissue regeneration is of great importance. In particular, the structure of the periodontal complex comprises two mineralized tissues (bone and cementum) in close proximity to an unmineralized tissue (PDL), and we were able to take advantage of the differences in multilineage differentiation potential between PDLSCs and JBMSCs. These findings indicated that a combination of different seeded cell types might be a more effective strategy^4^. In terms of gene expression, JBMSCs (P2) exhibited high expression of stemness-related genes (Nanog); adhesion-related genes (Fn1 and Lama1); and calcification-related genes (Col1a1, Col3a1, Periostin, Ibsp, Spp1 and Runx2) but not Notch1 or Cemp1. Therefore, we characterized the distinctions in molecular expression profiles between PDLSCs and JBMSCs, and these differences in gene regulation may induce the activation of specific signaling pathways, resulting in divergences in proliferation and differentiation between these two stem cell types^29^,^31^.

Second, we induced cells to form cell sheets for potentially more effective clinical applications. Cell sheet engineering has emerged as a novel approach to effectively deliver seeded cells while adequately preserving cell–cell junctions and the cellular microenvironment during tissue regeneration^19^, and these enhancements increase the efficiency of cell delivery and reduce the inflammatory reactions induced by foreign biomaterials. Iwata, T *et al*. determined that multilayered PDLSC sheets are useful for periodontal regeneration in a canine model^14^. Yang *et al*. showed that TDM induced and supported DFCs to develop new dentin pulp-like tissues and cementum-periodontal complexes in a nude mouse model^15^. In addition, Zhao developed a cell transplantation method that used PDLSC sheet fragments and PRF granules to enhance periodontal healing following avulsed tooth reimplantation in a canine model, and the results showed that the PDLSCs/PRF group achieved more effective periodontal healing, characterized by the regeneration of PDL-like tissues and a reduction of ankylosis and inflammation, than did the other tested groups^26^. Recent studies have shown that seeding cell sheets induces less immunogenicity than does seeding single cells, although the mechanism underlying this difference is unclear^31^. In traditional tissue engineering approaches, seeded cells are typically collected using trypsin, which degrades cell-to-cell junction proteins and the ECM, and this procedure might lead to cell loss and decrease cell survival^31^. In addition, by using our cell sheet technique, we could conveniently manipulate the transplantation of seeded cells, i.e., control the number, size, and location of the seeded cells. This technique also served as a simple and manageable method to combine seeded cells with other biomaterials for clinical use^6^,^31^.

To fabricate PDLSC and JBMSC cell sheets, high-density cells were cultured continuously for 2 weeks in complete medium containing 50 µg/mL of ascorbic acid, which has been shown to effectively promote the synthesis and deposition of a collagen matrix^14^. In this study, both the PDLSC and JBMSC sheets were closely arranged in a unidirectional or whirlpool-like pattern as shown by inverted microscopic observation and SEM, and this well-organized structure may optimize the function of these cells in developed periodontal tissue^32^. However, our observation of lateral sections via SEM and H&E staining showed that the PDLSC sheets contained multiple (3 to 7) layers of cells, secreted rich ECM and tended to form a tight network of collagen fibers that retained tight junctions. By contrast, JBMSC sheets contained fewer (2 to 4) layers of cells and secreted a limited amount of ECM. These differences may be attributed to the stronger proliferation ability of PDLSCs than JBMSCs and the tendency of PDLSCs to develop thicker collagen fibers^29^.

PDLSCs and JBMSCs exhibited very different mRNA expression profiles after forming cell sheets, and the expression of nearly every gene we evaluated displayed an increasing trend after cell sheet formation. For example, after sheet formation, both PDLSC and JBMSC sheets exhibited higher expression levels of periodontal tissue-specific genes (Col1a1 and Col3a1); calcification-related genes (Runx2, Ibsp, Spp1 and Bglap); and adhesion-related genes (Fn1 and Lama1) compared to the corresponding cells. These results implied that cell sheets may be superior to cultured cells for the construction of periodontal tissue because cell sheets exhibit a more favorable transcriptional profile^29^,^31^. In addition, we compared gene expression profiles between PDLSC and JBMSC sheets, and the results indicated that JBMSC sheets exhibited higher expression of calcification-related genes (Spp1 and Bglap), adhesion-related genes (Fn1 and Lama1), and periodontal tissue-specific genes (Col3a1 and Periostin) but lower expression of Cemp-1 than did PDLSC sheets. We found similar results for the protein expression level between these two types of stem cell sheets using western blot assays. These results indicated that the differential gene expression profiles of PDLSCs and JBMSCs continue from primary cells to cell sheets and that these differences influence the structure and function of newly formed tissues *in vivo*^29^. Based on immunohistochemical staining, both types of cell sheets displayed strong positive staining for FN1 and COL I; however, both PDLSC and JBMSC sheets demonstrated weakly positive staining for TNAP. COL I, the most abundant protein in ECM, serves as the structural support and binding partner for other ECM proteins, such as fibronectin. Therefore, COL I has been shown to promote the activation, migration, proliferation and differentiation of embedded stem cells^33^. In addition, the abundance of COL I in the examined samples indicated that both types of cell sheets support the formation of a natural, collagen-rich structure that can easily be fabricated into different shapes and sizes, thereby simplifying the process of combining seeded cells^31^,^34^. TNAP is an early marker of osteoblast differentiation, and the weakly positive staining for TNALP implied osteogenic differentiation potential in both JBMSC and PDLSC sheets.

Third, we used PRF as a biodegradable scaffold to support, nourish and sustain cell sheets. As a 3-D fibrin network that releases multiple growth factors, PRF has been widely used in oral and plastic surgery^35^,^36^. PRF has also been used as a scaffold adjuvant in tissue engineering because it can enhance cell proliferation and ECM metabolism in various connective tissues. Our ELISA results showed that high levels of growth factors were continuously released from α granules of PRF for at least seven days^26^. PRF has several advantageous properties, such as high efficiency, one-step preparation and the provision of natural blood products in the absence of anticoagulants^36^. PRF has been widely reported to promote the proliferation ability of gingival fibroblasts, osteoblasts, JBMSCs and PDL cells but not epithelial cells due to the release of a set of growth factors that include platelet-derived growth factor (PDGF), basic fibroblast growth factor (bFGF), and insulin-like growth factor-1 (IGF-1). Therefore, PRF has been shown to either promote cell proliferation or increase ECM synthesis^37^. Due to its influence on differentiation, TGF-β plays an important role in embryonic development, cell differentiation and tissue maturity. IGF-I exerts an additive effect when combined with TGF-β^38^. In this study, PRF was designed to integrate with cell sheets to favorably deliver, support and nourish cell sheets in a specific area of periodontal destruction, thereby enhancing the quality of periodontal regeneration *in vivo*.

To confirm the validity of the combined application of PDLSC sheets, JBMSC sheets and PRF, we designed a new strategy for the construction of periodontal tissue in subcutaneous pockets of nude mice. As shown in Fig. 4, PDLSC and JBMSC sheets were used for PDL and bone engineering, respectively, and PRF membranes were inserted between the cell sheets to act as an absorbable biomaterial and provide various growth factors^19^. Previous studies have shown that PRF functions as a complex regenerative scaffold to promote both surrounding PDL regeneration and tissue-specific alveolar bone production via progenitor-specific mechanisms^27^,^39^. In addition, a subcutaneous implantation study indicated that PRF could readily integrate with surrounding tissues and be partially replaced with collagen fibers 2 weeks after implantation^27^. The microenvironmental niche is known to play a crucial role in optimal tissue regeneration. In this study, TDM was used to simulate the microenvironment of dentin tissue because TDM has been shown to induce the formation of cementum-/PDL-like tissues from PDLSCs or PDLSC sheets^8^. By contrast, the HA/TCP framework was employed to simulate alveolar bone tissue because this framework contains abundant levels of HA, a common component of mature bone tissues. The HA/TCP framework can also provide a microenvironment that supports osteoinduction to promote bone regeneration^6^. Ring-shaped interspaces^15^ were formed after inserting TDM into the HA/TCP framework, and these voids provided space and a microenvironment for the activities of the transplanted cell sheets and PRF. To explore the tissue-specific regenerative potential of PDLSC and JBMSC sheets, we designed three PRF-cell sheet constructs for *in vivo* transplantation, namely PDLSC sheets/PRF/PDLSC sheets, JBMSC sheets/PRF/JBMSC sheets, and PDLSC sheets/PRF/JBMSC sheets.

Eight weeks after subcutaneous implantation into nude mice, collagen fibers were predominantly formed when PDLSC sheets were used. Although it is widely accepted that PDL cells or cell sheets could be induced into mineralization toward cementum formation by the microenvironment of TDM^8^,^15^,^16^, we did not detect any obvious cementum-like structures. This may be because mineralized cementum and dentin are very similar in terms of physical location, composition, and percent mineralization, and therefore, the use of thick sections here prevented the demonstration of a cementum layer^40^. By contrast, only JBMSC sheets were able to form bone-like tissue regardless of the scaffolding materials (TDM or HA/TCP). These findings further indicated that specific phenotypic stem cells tend to develop new tissues that match their origin in terms of structure and function, as reported previously^17^,^18^. From the data obtained in this study, the design based on PDLSC sheets/PRF/JBMSC sheets appears to be the most promising strategy to achieve the regeneration of multiple periodontal tissues.

Although we identified some PDL-like fibers that were not inserted and some bone-like tissues that were not well characterized, we did confirm that PDL- and bone-like structures can be regenerated based on PDLSC sheets/PRF/JBMSC sheets in a nude mouse model. Unfortunately, we cannot provide exact evidence of cementum formation on the TDM surface because of the thick sections we used, which can maintain the complete HA/TCP and dentin framework as well as the newly formed tissues. In future studies, thin sections (e.g., 5 to 6 microns) or SEM will be used to identify potential cementum regeneration. In addition, the strategy of PDLSC sheet/PRF/JBMSC sheet transplantation in areas of periodontal tissue destruction in periodontitis patients must be investigated in future studies. Studies must also be conducted to assess whether the regenerated periodontal complex apparatus exhibits a mature structure and complete function to support and resist occlusal force^41^. Although all of the transplanted biomaterials (PDLSC sheets, JBMSC sheets and PRF) used in this study could be acquired from autologously donated materials, the potential for immune rejection following the *in vitro* expansion and manipulation of these materials must be explored further^42^.

## Conclusion

In this study, we isolated two specific phenotypic stem cell types, PDLSCs and JBMSCs, for use as seeded cells and compared the differentiation tendencies of these cell types *in vitro* and *in vivo*. The results indicated that PDLSCs tended to produce PDL-like tissues, and JBMSCs tended to generate bone-like tissues. When each cell type was induced to form cell sheets, these differentiation tendencies persisted. To improve the therapeutic efficiency of cell sheets, we used PRF comprising a 3-D fibrin network and various growth factors to support, sustain and nourish the cell sheets in the space between alveolar bone and the root surface. For the *in vivo* implantation of cell sheets and PRF in nude mice, we designed TDM and HA/TCP frameworks to simulate the interfaces of the root surface and alveolar bone, respectively. Finally, 8 weeks after implantation, PDL- and bone-like structures were successfully produced from a PDLSC sheet/PRF/JBMSC sheet composite. Although further evidence of well-organized PDL and bone tissues and cementum regeneration is needed, this cell transplantation method may be a potential approach for periodontal tissue engineering and regeneration.

## Materials and Methods

### Isolation and characterization of PDLSCs and JBMSCs

#### Isolation of stem cells

Human PDLSCs were isolated and cultured based on approved guidelines^17^. Briefly, the PDL was isolated from extracted second premolars of orthodontic patients (18 to 23 years of age, n = 3). All patients provided written informed consent, and the IRB from the Stomatological Hospital of the Fourth Military Medical University approved the experiment. The PDL tissues in the middle portion of the root surfaces were carefully scraped, washed repeatedly with PBS, and cut into small blocks (approximately 1 mm^3^). The tissue blocks were digested in 5 mL of α-MEM (Hyclone, MA, USA) containing 1% collagenase type I and 1% dispase (both from Sigma-Aldrich, St. Louis, MO, USA) for 40 min at 37 °C. The digested tissues were then transferred to 3.5-cm diameter culture dishes (Corning, Lowell, MA, USA) and cultured in a complete medium comprising α-MEM supplemented with 15% fetal bovine serum (FBS, Hyclone); 0.292 mg/mL of glutamine (Sigma-Aldrich, St. Louis, MO, USA); 100 mM of L-ascorbic acid (Sigma-Aldrich); 100 U/mL of penicillin (Sigma-Aldrich); and 100 mg/mL of streptomycin (Sigma-Aldrich). Finally, the dishes were incubated in a humidified atmosphere at 37 °C in 5% CO_2_, and the culture media were replaced every 3 days until the cells had successfully migrated from the PDL tissue blocks. As described elsewhere, the limiting dilution technique was used to obtain single cell-derived clones (P0). PDLSCs at passages P3-P5 were used in subsequent experiments.

To isolate the JBMSCs^43^, an approved guideline was adopted. Briefly, fresh cancellous bone fragments and blood were obtained from three orthognathic patients (20 to 26 years of age) via mandibular angle resection. All patients provided written informed consent, and the IRB from the Stomatological Hospital of the Fourth Military Medical University approved the experiment. The fragments were quickly transferred to a clean bench. A syringe was used to pour α-MEM repeatedly and gently over the fragments until they displayed a white appearance. The mixed cell types in medium were then centrifuged, resuspended and seeded in a six-well plate (Corning) containing complete medium. The cells were cultured as described above until clones of JBMSCs formed and reached 90% confluence. In the same way, the limiting dilution technique was used to obtain single cell-derived clones (P0). JBMSCs at passages P3-P5 were used in subsequent experiments.

#### Colony formation assay

CFU-F assays were employed to determine the colony-forming abilities of the stem cells. Briefly, human PDLSCs (P3) or JBMSCs (P3) were seeded in 10-cm diameter culture dishes at a density of 1 × 10^3^ cells per dish and cultured in complete medium. After 12 days of incubation, the cells were fixed in 4% paraformaldehyde and stained with 0.1% crystal violet (Sigma-Aldrich) for 15 min. The dishes were then washed twice with PBS and observed under an inverted microscope. Aggregates of 50 or more cells were considered colonies and were included in the final statistical analysis; aggregates of fewer cells were excluded from the analysis.

#### Cell proliferation assay

The CCK-8 (Beyotime Institute of Biotechnology, Jiangsu, China) assay was used to quantitatively evaluate the number of proliferating cells during a 7-day culture period in complete medium. Briefly, PDLSCs (P3) and JBMSCs (P3) were plated in 96-well dishes (Nunc, Thermo, Denmark) at a density of 2 × 10^3^ cells per well, and the culture medium was replaced every 3 days. At a proscribed time every day, the primary culture medium was removed, and 2 mL of fresh complete medium was mixed with 200 μL of CCK-8 reagent using a pipette tip. An aliquot (150 μL) of this mixed solution was then added to each well. Following incubation at 37 °C for 3 h, the incubated media were transferred to a new 96-well plate, and an absorbance at 450 nm was measured using a spectrophotometer (ELx800, BioTek Instruments Inc., Highland Park, IL, USA). As a background control, the same volume of the mixed solution in the absence of cells was incubated and subjected to the same process in parallel. Three parallel experiments were performed for each group for the proliferation assay.

#### Flow cytometric analysis of cell surface markers

Flow cytometric analysis was used to determine the expression of cell surface markers by PDLSCs and JBMSCs (P3) as described previously^17^. Briefly, after each cell line reached 90% confluence in 10-cm dishes, the cells were digested with trypsin and washed twice with PBS. The cell suspensions in PBS were then divided into sterile microtubes and incubated in 0.1% antibodies against human STRO-1 (BD Bioscience, San Jose, CA, USA); CD146 (eBioscience, San Diego, CA, USA); CD29 (eBioscience); CD34 (BioLegend, San Diego, CA, USA); and CD45 (BioLegend) at 37 °C in the dark for 40 min; cell suspensions in PBS without any antibodies served as controls. The cells were then washed three times with PBS to remove excess antibodies. Finally, the cells were resuspended in 300 μL of PBS supplemented with 3% FBS and analyzed via fluorescence-activated cell sorting using an FACSVantage Cell Sorter (Becton Dickinson, Mountain View, CA, USA).

#### *In vitro* osteogenic/adipogenic differentiation assays

PDLSCs (P3) and JBMSCs (P3) were seeded in six-well dishes at a density of 5 × 10^5^ cells per well and incubated in complete medium until they reached 90% confluence. To assess the osteogenic ability of each cell type, the osteoinduction medium (complete medium supplemented with 1.8 mM of KH_2_PO_4_, 50 μg/mL of ascorbic acid and 10 nM of dexamethasone) was replaced at 3-day intervals. After 4 weeks of osteogenic induction, the cells were fixed in 4% paraformaldehyde and stained with Alizarin Red S staining solution (Sigma-Aldrich) for 3 min at room temperature. The dishes were then washed twice with PBS, dried at room temperature and observed under a microscope. Finally, the mineralized nodules in each cell line were dissolved in 2% cetylpyridinium chloride, and the OD at 560 nm was measured using a spectrophotometer, followed by statistical analysis. To assess adipogenesis, the adipogenic medium (complete medium supplemented with 100 nM of dexamethasone, 10 μg/mL of insulin, 0.5 mM of 3-isobutyl-1-methylxanthine and 50 mM of indomethacin) was replaced every three days. After two weeks, the cells were fixed in 4% paraformaldehyde and stained with Oil Red O (Sigma–Aldrich) staining solution for 15 min. The dishes were then washed twice with PBS and observed under a microscope. Finally, lipid droplets were dissolved in isopropanol, and the OD at 560 nm was quantified using a spectrophotometer, followed by statistical analysis.

#### *In vivo* osteogenic differentiation assays

The animal experiments were performed according to the guidelines of the committee for animal experiments of the Fourth Military Medical University, and the Institutional Review Board for Human Subjects Research of the Fourth Military Medical University approved the experimental protocols. For subcutaneous implantation of *in vitro*-expanded PDLSCs (P4) and JBMSCs (P4), 2.0 × 10^6^ culture-expanded cells were suspended in 0.5 mL of complete medium mixed with 40 mg of HA/TCP (National Engineering Research Center for Biomaterials of Sichuan University, Chengdu, China) and incubated for 90 min at 37 °C. After a short centrifugation to remove the supernatant, the cell/carrier mixtures were immediately implanted into the dorsum of nude mice. Eight weeks after implantation, all nude mice were sacrificed. Then, 3-D reconstruction images of the grafts were obtained using micro-CT (Enhanced Vision Systems Model MS-8 *In Vitro* Micro-CT Scanner; GE Healthcare, London, Ontario, Canada), and the equivalent bone density of each sample was calculated and used for statistical analysis. The harvested samples were then fixed with 4% paraformaldehyde for 48 h at 4 °C, demineralized with 10% EDTA (pH 6.9) at room temperature for 2 weeks, and embedded in paraffin. The paraffinized sections were then stained with H&E to observe the regenerated tissue.

#### Gene expression profiles of PDLSCs and JBMSCs

Real-time PCR was used to determine the differences in gene expression between PDLSCs (P2) and JBMSCs (P2). Briefly, total RNA was extracted from the cells using TRIzol reagent (Invitrogen, Carlsbad, CA, USA), and cDNA was synthesized using total RNA samples (Takara Bio, Otsu, Japan). cDNA was then diluted ten-fold and mixed with primers (Sangon Biotechnology Co., Shanghai, China), double-distilled water and SYBR Green Master Mix (Roche). Finally, the mixed solution was subjected to real-time PCR procedures using a CFX Connect™ real-time PCR Detection System (Bio-Rad, Hercules, CA, USA). The following genes were monitored: Fn1, Lama1, Notch1, Nanog, Col1a1, Col3a1, Periostin, Runx2, Ibsp, Spp1, Bglap, Cemp1, Hacd1 and Gapdh (Table 2). The relative expression levels of the target genes were normalized to the expression level of the Gapdh gene.

### Cell sheet formation and characterization

#### Induction of cell sheet formation

Cell sheets were produced as described previously^14^. PDLSCs (P3) and JBMSCs (P3) were separately seeded in 6-well plates (5 × 10^4^ cells/well) and cultured in complete medium. After 24 h, the medium was replaced with a sheet-inducing medium comprising complete medium supplemented with 50 μg/mL of ascorbic acid; this medium was replaced every 3 days. After 14 days of incubation, the formed cell sheets were observed under an inverted microscope and detached using ophthalmic forceps.

#### H&E staining

For H&E staining, the detached cell sheets were fixed in 4% paraformaldehyde for 30 min, embedded in paraffin and sliced into 5-μm sections. The stained sections were then observed photographically.

#### SEM observation

For SEM observation, the detached cell sheets were fixed in 70% ethanol at 4 °C for 30 min, dehydrated using a graded ethanol series, and dried. The surface and lateral section topographies of the cell sheets were observed using SEM (Hitachi S-4300; EIKO Engineering, Tokyo, Japan), and the sheet thickness was quantitatively measured from the photographs of the sections using Photoshop 7.0.

#### Immunohistochemical staining

For immunohistochemical staining, paraffinized sections (5 μm) of PDLSC or JBMSC sheets were deparaffinized; blocked; and incubated for 1 h in the following primary antibodies: (1) anti-FN1 (1:200), (2) anti-COL I (1:200), and (3) anti-TNALP (1:200) (1:200, all from Santa Cruz Biotechnologies, Dallas, TX, USA). PBS was used in place of the primary antibodies for negative controls. The sections were then incubated for 45 min in biotinylated secondary antibodies (1:1000) purchased from ZSGB BIO (Peking, China). Each experiment was repeated in triplicate.

#### Real-time PCR analysis after sheet formation

For real-time PCR analysis, total RNA was isolated from PDLSCs (P2), JBMSCs (P2), PDLSC sheets and JBMSC sheets using TRIzol reagent. The cDNA synthesis and real-time PCR procedures were performed sequentially as described above. Finally, the relative expression levels of the target genes were normalized to the expression level of the Gapdh gene for each group.

#### Western blot analysis after sheet formation

For western blot analysis, PDLSC and JBMSC sheets were lysed in lysis buffer (Sigma-Aldrich) supplemented with 1 mM of PMSF (Roche, Basel, Switzerland). The total protein concentrations were then measured using a bicinchoninic acid protein assay kit (Beyotime). Next, 40 μg of cell lysates were added to each lane of 10% SDS-PAGE gels, separated based on molecular weight, and electrotransferred to polyvinylidene fluoride membranes (Millipore, Billerica, MA, USA). After blocking with 5% non-fat milk for 2 h, the membranes were incubated in specific primary antibodies against COL I, COL III, OPN, OCN, CEMP1 and CAP (all from Santa Cruz Biotechnologies) overnight at 4 °C. The membranes were then incubated in a horseradish peroxidase-conjugated anti-rabbit or anti-mouse secondary antibody (CoWin Biotech Co., Ltd., Beijing, China) for 1 h at room temperature. The membranes were developed using a Western Light Chemiluminescent Detection System (Peiqing, Shanghai, China). All assays were repeated in triplicate.

### Regeneration of periodontal tissue *in vivo*

#### PRF preparation

First, 100 mL of venous blood was collected from volunteers (20 to 25 years old, n = 3) based on approved guidelines^29^. All patients provided written informed consent, and the IRB from the Stomatological Hospital of the Fourth Military Medical University approved the research project. Briefly, 10 mL of blood was rapidly withdrawn from the median cubital vein using a sterilized 10-mL syringe, transferred to test tubes without anticoagulant, and immediately centrifuged for 12 min at 400 g. The upper platelet-poor plasma layer was then discarded, and the fibrin clot was separated from the red blood corpuscle (RBC) layer at the bottom using scissors. An area of the RBC layer of at least 1 mm was carefully retained due to the concentrations of leukocytes and platelets at the junction zone. Several sterile dry gauzes were used to softly compress the fibrin clot between the palms to squeeze the fluids out of the clot. A resistant PRF membrane was obtained and used for subsequent experiments.

#### Quantification of the levels of growth factors in PRF

The levels of crucial growth factors contained in one complete PRF membrane, i.e. TGF-β1, PDGF-AB, vascular endothelial growth factor (VEGF), IGF-1 and epidermal growth factor (EGF), were quantified using ELISA kits (R&D Systems, Shanghai, China) based on the manufacturer’s instructions. Briefly, the PRF membrane was placed in a 3.5-cm diameter culture dish and incubated in 2.5 mL of PBS (Hyclone) for 7 days in a cell incubator. The entire PBS suspension was collected at a fixed time every day, and an equal volume of PBS was applied for sequential incubation. All collected samples were transferred to sterile 2-mL microtubes and stored at −80 °C. Finally, the samples were simultaneously assessed, and the data were expressed as the mean ± standard deviation and statistically analyzed.

#### Preparation of human TDM and HA/TCP frameworks

To simulate the dentin components used for PDL tissue attachment, human TDM was prepared as described previously^15^. Briefly, premolar teeth were extracted for orthodontic reasons at the Stomatology Hospital of the Fourth Military Medical University. All patients provided written informed consent, and the IRB from the Stomatological Hospital of the Fourth Military Medical University approved the research project. The root surfaces of the collected teeth were carefully cleaned and scraped with a curette to remove periodontal tissue. The crowns at the cemento-enamel junction were then rapidly removed using a high-speed dental turbine handpiece, and pre-dentin and dental pulp tissues were simultaneously discarded mechanically. The TDM was then sequentially treated with 17% EDTA (Sigma, USA) for 5 min, 10% EDTA for 5 min and 5% EDTA for 10 min. The TDM was then mechanically cleaned twice in double-distilled water using an ultrasonic cleaner. Human TDM samples were air-dried and irradiated with ultraviolet light overnight on a super-clean bench; transferred to a sterile PBS solution containing 100 U/mL of penicillin (Hyclone, USA) and 100 mg/mL of streptomycin (Hyclone, USA) for 24 h; washed in sterile deionized water for 10 min in an ultrasonic cleaner; and stored in α-MEM at 4 °C.

To simulate the mineralized microenvironment of the alveolar bone, we constructed HA/TCP frameworks^44^ with inner contours that matched the outer conical contours of the TDM. Holes larger than the human TDM were drilled inside the HA/TCP frameworks to ensure that there was sufficient space (0.5 to 1 mm) between these materials for the periodontium to regenerate from cell sheets and PRF. Finally, the HA/TCP frameworks were irradiated with cobalt 60 and stored in α-MEM at 4 °C.

#### SEM observation

To characterize the ultrastructure of the PRF membrane, the obtained PRF membrane fabricated as described above was fixed in 2.5% glutaraldehyde at 4 °C for 30 min, dehydrated using a graded ethanol series and freeze-dried. The surface topography was observed via SEM (Hitachi S-4300; EIKO Engineering, Tokyo, Japan).

To characterize the ultrastructure of the TDM and HA/TCP frameworks, the biomaterials were processed as described above, dried, sputtered, and observed via SEM.

#### Regeneration of tooth root *in vivo*

To evaluate the periodontal regeneration potential of PDLSC and JBMSC sheets *in vivo*, the cell sheets and TDM and HA/TCP frameworks were transplanted into the subcutaneous dorsa of nude mice. The animal experiments were performed according to the guidelines of the committee for animal experiments of the Fourth Military Medical University, and the Institutional Review Board for Human Subjects Research of the Fourth Military Medical University approved the experimental protocols. Briefly, after 2 weeks of culture in 10-cm dishes, mature cell sheets were carefully extracted. The treated dentin matrix was carefully wrapped with at least three layers of PDLSC sheets, JBMSC sheets or PRF membrane and placed in the inactivated HA/TCP frameworks. The above biomaterials were randomly divided into three groups according to their composition: group 1: PDLSC sheets/PRF/PDLSC sheets (order from the TDM side to the HA/TCP side); group 2: JBMSC sheets/PRF/JBMSC sheets; and group 3: PDLSC sheets/PRF/JBMSC sheets. These complexes were then incubated in complete medium containing 50 μg/mL of ascorbic acid at 37 °C for 90 min to facilitate stable adhesion. Male 6-week-old nude mice were obtained from the animal center of the Fourth Military Medical University. The cell sheets and biomaterial complexes (n = 5) were implanted into the subcutaneous dorsa of immunodeficient mice under deep anesthesia via an intraperitoneal injection of 10% chloral hydrate, and the incisions for material implantation were carefully sutured.

After eight weeks, samples were acquired and fixed in 4% paraformaldehyde for 2 days at 4 °C. The specimens were then embedded in plastic and sectioned (20- to 30-μm thick sections) using a hard tissue-slicing method. The sections were stained with H&E or Masson’s trichrome (Sigma-Aldrich, St. Louis, MO, USA). For immunohistochemical staining, the plastic sections were blocked and incubated for 1 h in an anti-human COL-I primary antibody (1:200, Santa Cruz Biotechnologies, Dallas, TX, USA). PBS was applied instead of the primary antibody for negative controls. The specimens were then incubated for 45 min in biotinylated secondary antibodies (1:1000) purchased from ZSGB BIO (Peking, China). The experiments were repeated in triplicate. All histological observations were performed using a light microscope (BX50, Olympus Optical, Japan), and photographs were taken (DP25, Olympus).

### Statistical analysis

The results were collected and analyzed using SPSS 17.0 software (SPSS, IBM, Armonk, NY, USA). All values were expressed as the mean ± standard deviation, and the level of significance was determined using one-way ANOVAs and Student-Newman-Keuls post hoc tests. *P* < 0.05 was considered statistically significant.

## Additional Information

**How to cite this article**: Wang, Z.-S. *et al*. The use of platelet-rich fibrin combined with periodontal ligament and jaw bone mesenchymal stem cell sheets for periodontal tissue engineering. *Sci. Rep.* **6**, 28126; doi: 10.1038/srep28126 (2016).

## Figures and Tables

**Table 1:** The levels of growth factors released from one complete PRF membrane over 7 days as determined by ELISA.

PRF releasate	TGF-β1 (pg)	PDGF-AB (pg)	IGF- 1 (pg)	VEGF (pg)	EGF (pg)
D1	1091.46 ± 95.04	1023.49 ± 83.67	6508.67 ± 438.46	756.03 ± 67.25	317.22 ± 21.36
D2	990.857 ± 89.066	679.19 ± 44.23	1487.63 ± 107.27	276.43 ± 51.76	169.67 ± 13.12
D3	877.28 ± 64.84	600.20 ± 33.66	898.04 ± 88.91	179.10 ± 17.25	137.21 ± 10.89
D4	794.09 ± 53.97	389.22 ± 41.69	790.78 ± 64.98	118.49 ± 13.38	69.15 ± 5.10
D5	654.70 ± 76.95	346.44 ± 33.42	332.38 ± 27.19	58.32 ± 9.11	27.24 ± 2.88
D6	184.35 ± 32.55	144.59 ± 11.84	271.42 ± 17.18	46.16 ± 4.88	23.29 ± 3.32
D7	56.943 ± 4.16	74.46 ± 3.92	190.21 ± 35.88	40.67 ± 3.55	19.19 ± 3.44

Abbreviations: PRF, platelet-rich fibrin; TGF-β1, transforming growth factor-β1; PDGF-AB, platelet-derived growth factor; IGF-1, insulin-like growth factor-1; VEGF, vascular endothelial growth factor; EGF, epidermal growth factor.

**Table 2:** Target cDNA primer sequences used for real-time PCR.

**Fn1**
Forward: 5′-CGGTGGCTGTCAGTCAAAG-3′
Reverse: 5′-AAACCTCGGCTTCCTCCATAA-3′
**Lama1**
Forward: 5′-ACTGGACTCACCTACGCCAAC-3′
Reverse: 5′-GGGAGCACACTGGTCACATTT-3′
**Notch1**
Forward: 5′-CGAACCAATACAACCCTCTGC-3′
Reverse: 5′-CTGGTAGCTCATCATCTGGGACA-3′
**Nanog**
Forward: 5′-TTTGTGGGCCTGAAGAAAACT-3′
Reverse: 5′- AGGGCTGTCCTGAATAAGCAG-3′
**Col1a1**
Forward: 5′-GAGGGCCAAGACGAAGACATC-3′
Reverse: 5′-CAGATCACGTCATCGCACAAC-3′
**Col3a1**
Forward: 5′-GGAGCTGGCTACTTCTCGC-3′
Reverse: 5′-GGGAACATCCTCCTTCAACAG-3′
**Periostin**
Forward: 5′-TGGAGAAAGGGAGTAAGCAAGG-3′
Reverse: 5′-TTCAAGTAGGCTGAGGAAGGTG-3′
**Runx2**
Forward: 5′-TGGTTACTGTCATGGCGGGTA-3′
Reverse: 5′-TCTCAGATCGTTGAACCTTGCTA-3′
**Ibsp**
Forward: 5′-GATTTCCAGTTCAGGGCAGTAG-3′
Reverse: 5′- CCCAGTGTTGTAGCAGAAAGTG-3′
**Spp1**
Forward: 5′-CAGTTGTCCCCACAGTAGACAC-3′
Reverse: 5′-GTGATGTCCTCGTCTGTAGCATC-3′
**Bglap**
Forward: 5′-CCCAGGCGCTACCTGTATCAA-3′
Reverse: 5′-GGTCAGCCAACTCGTCACAGTC-3′
**Cemp1**
Forward: 5′-AACACATCGGCTGAGAACCTCAC-3′
Reverse: 5′-GGATACCCACCTCTGCCTTGAC-3′
**Hacd1**
Forward: 5′- CCTGGCTCACCTTCTACGAC-3′
Reverse: 5′-CCTCAAGCAAGGCAAATGTC-3′
**Gapdh** (housekeeping gene)
Forward: 5′-CTTTGGTATCGTGGAAGGACTC-3′
Reverse: 5′-GTAGAGGCAGGGATGATGTTCT′-3′

Abbreviations: Fn1, fibronectin 1; Lama1, laminin, alpha 1; Col1a1, collagen type I, alpha 1; Col3a1, collagen type III, alpha 1; Runx2, runt-related transcription factor 2; Ibsp, integrin binding sialoprotein; Spp1, secreted phosphoprotein 1; Bglap, bone gamma-carboxyglutamate protein; Cemp1, cementum protein 1; Hacd1, 3-hydroxyacyl-CoA dehydratase 1.
